# Instruments for Outcome Evaluation of Specific Domains in Primary Sjögren’s Syndrome

**DOI:** 10.3390/biom11070953

**Published:** 2021-06-28

**Authors:** Nicoletta Del Papa, Antonina Minniti, Wanda Maglione, Francesca Pignataro, Roberto Caporali, Claudio Vitali

**Affiliations:** 1 Department of Rheumatology, Azienda Socio Sanitaria Territoriale Gaetano Pini—Centro Traumatologico Ortopedico, 20122 Milano, Italy; antonina.minniti@asst-pini-cto.it (A.M.); wanda.maglione@asst-pini-cto.it (W.M.); francesca.pignataro@asst-pini-cto.it (F.P.); roberto.caporali@unimi.it (R.C.); 2 Research Center for Adult and Pediatric Rheumatic Diseases, Department of Clinical Sciences and Community Health, Università degli Studi di Milano, 20122 Milano, Italy; 3 Rheumatology Outpatient Clinics, Mater Domini Humanitas Hospital, 21053 Castellanza, Italy; c.vitali@yahoo.it

**Keywords:** Sjogren’s syndrome, outcome measures, sicca symptoms, disease activity

## Abstract

Primary Sjögren’s syndrome (pSS) is a systemic autoimmune disorder characterized by very heterogeneous features. The spectrum of this disorder may vary from benign but disabling symptoms such as dryness, due to lachrymal and salivary involvement, pain and fatigue, to systemic, potentially severe, manifestations that may involve any organ. In recent decades, the arrival of biotechnological therapy has offered new opportunities for the treatment of this—until now—orphan disease. Currently, the possible use of these new drugs in therapeutic trials has made it necessary to have reliable outcome measures to evaluate their efficacy in this disease. A great effort has been made in multicenter, often multinational, studies to develop and validate instruments capable of assessing the different disease-related features. The adoption in therapeutic trials of the newly developed outcome measures aimed at assessing systemic features and patient reported symptoms has often yielded disappointing results. These negative data have been ascribed, on the one hand, to the trial design not being completely appropriate, and, on the other hand, to the fact that a single instrument may be not sufficient to cover the great clinical heterogeneity of the disease features. There is now growing belief that composite end points that include instruments that are able to assess the various aspects of the disease may be more properly and successfully used in future therapeutic trials.

## 1. Introduction

Primary Sjögren’s syndrome (pSS) is a systemic autoimmune disease that mainly affects the exocrine glands and commonly presents as persistent dryness of the mouth and eyes as a consequence of functional impairment of the salivary and lachrymal glands [1]. However, other exocrine glands and non-exocrine epithelial tissues may be involved. Therefore, the term “autoimmune epithelitis” has been proposed to define the disorder [2]. In fact, the histologic hallmark of SS is lymphocytic infiltration of the involved tissues (exocrine and non-exocrine epithelia). The inflammatory infiltrates can comprise activated T cells, while in other cases B cells are predominant [3]. Persistent B-cell activation and proliferation is considered to be an important feature of the disease, which may result in the development of B-cell lymphoma [4,5].

In a large number of patients, the clinical course of SS is slowly progressive and is commonly characterized by sicca symptoms, fatigue, and widespread pain. Conversely, from 30% to 50% of patients may develop more evident flares of the disease, which are characterized by the presence or sudden appearance of extraglandular systemic features. These are Raynaud’s phenomenon, arthritis, vasculitis, interstitial lung disease, leukopenia, and peripheral neuropathy, while glomerulonephritis and central nervous system involvement are more rarely recorded [6].

For many years pSS has been considered as an orphan disease since no treatment has proved to be capable of modifying its natural course. The therapeutic approach has been based on symptomatic replacement or stimulation of glandular secretions, using tear and saliva substitutes and muscarinic agents. Extraglandular features have been empirically treated with corticosteroids and immunosuppressive agents similar to what is commonly done for similar clinical manifestations in patients with other systemic autoimmune diseases [7]. The availability of new biotechnological agents that are potentially able to target molecules and cells that appear to play a relevant role in the pathogenesis of pSS has opened a new era in the management of patients with this disease [8]. Contemporaneously, the arrival of these new treatments has raised the issue of the need for reliable disease-specific outcome measures and tools that could be effective in evaluating symptoms, single organ involvement, and general disease status entities in patients with pSS both in clinical trials and clinical practice [9].

Two important and different entities are classically scored by disease status indices, i.e., activity and damage. Conceptually, activity implies reversibility of the process and is usually characterized by inflammatory changes in various organs or systems. Damage represents the component of the disease process that is irreversible and can be defined as a permanent loss of function or by radiographically or histologically evident structural alterations of the involved organ or system.

Severity represents the stratification of the reversible and irreversible pathologic abnormalities caused by disease in a given organ or tissue and of the burden of these changes in a given individual. In other words, a disease manifestation, independent of the fact that it can be a consequence of an active phase of the disease or related to stable damage, is commonly considered severe when it can be fatal for the patient, cause important disability, or is particularly resistant to treatment [10].

A critical reappraisal of the instruments presently available to measure outcome in this disorder was carried out in the present review, giving particular attention to their intrinsic validity, reliability, sensitivity to change, and feasibility. Furthermore, for some of these instruments, some criticisms that have emerged during their use specifically in clinical trials are also discussed.

## 2. Measurement of Activity and Damage in pSS

Disease status indices should have the required clinometric and psychometric properties [10,11,12]. First, they should demonstrate their validity as instruments. The minimum requirement is to have face validity, which can be simply defined as the capability of measuring what they are designed to measure. Furthermore, they should have construct and content validity. To have construct validity, the scoring system should correlate with an external criterion of the state. The physician’s global assessment (PhGA) is commonly used for this purpose, and it is thus considered as the gold standard, although there are data showing that the PhGA may be inaccurate [13], particularly when made by differing observers with different cultural backgrounds and knowledge regarding the specific disorder. In addition, to have content validity a disease status index should cover all the relevant possible variables in the clinical expression of the disease. Reliability is another essential property for instruments devoted to measuring different clinical status entities. Generally speaking, reliability can be defined as the random error of a measurement system. If this error is small, the measure can be considered reliable. Inter-observer reliability (stability of the measure when used by different operators) and intrarater reliability (stability of the scale when used by the same operator on subsequent occasions) should both be satisfied if an index is to be considered reliable. To obtain a high level of reliability, the standard of data collection and the rules for data recording should be optimized [14]. Finally, disease status criteria, and particularly those devoted to measuring a reversible entity, as disease activity, should demonstrate a high level of sensitivity to any change in the clinical course, or at least be able to appreciate important clinical differences in patients’ status [15]. For this purpose, the concepts of minimal significant change and response criteria should be precisely defined.

## 3. Systemic Disease Activity Measurements

Specific instrument have been developed to measure disease activity in patients with systemic manifestations. Two indices derived from two national studies carried out in Italy (SSDAI, SS Disease Activity Index) [16] and the United Kingdom (SCAI, Sjögren’s Clinical Activity Index) [17] have been proposed to assess disease activity in patients with pSS.

The first one was developed by an Italian joint effort aimed at defining activity criteria by using the same methodology followed to develop the ECLAM (European Consensus Lupus Activity Measurement) index [18]. Data collected on a total of 206 patients recruited in 12 Italian centers were used to build a multivariate model predictive of disease activity. The PhGA of the observed grade of activity scored from 0 to 10 represented the dependent variables of the model. The weight of any variable included in the scale was derived from the coefficient of each variable in the model. In its final version, the SSDAI included only 15 items [16].

The data of patients who demonstrated a significant level of activity at the first observation time were collected during the second clinical observation 3 months later. This was done to assess the variation of the level of disease activity over time. Sensitivity to change of the SSDAI was then assessed by measuring the correlation between the time variation of the scores given by the observers over time and that of the scores recalculated by applying the constructed index [16]. In summary, the SSDAI had the benefit of simplicity, showed a good construct validity and sensitivity to change, but lacked content validity since some rare but severe manifestations of pSS were not included in the index.

Contemporaneously with the Italian group, a British group developed and validated an activity index to assess systemic features of pSS [17] based on a modified version of the previously developed BILAG (British Island Lupus Activity Group) index for systemic lupus [19].

The stratification of the domains included in the scale is performed according to the rater’s intention to treat. SCAI is made up of a nine-domain structure. Construct validity of the SCAI was also proved by measuring the correlation of this scale with the PhGA, while its sensitivity to change was tested by comparing SCAI-derived flares with physician-defined disease flares [17]. In comparison with the SSDAI, the SCAI has a more complete content validity, but its rating is so complicated that it is very difficult to use in clinical practice. Similar to the SSDAI, the SCAI has been constructed from a limited cohort of patients collected on a national basis.

The SSDAI and SCAI represented the first tentative approaches to assess disease activity in pSS. The fact that these indices have been developed in a limited number of patients and in single countries induced a group of European experts to join their efforts in a multinational initiative, under the patronage and the support of the European League Against Rheumatism (EULAR), to develop two separate indices aimed at assessing two different facets of the disease, i.e., the ESSDAI (European SS Disease Activity Index) for systemic features [20] and (the ESSPRI, European SS Patient-Reported Index) [21] for patients’ symptoms.

### 3.1. ESSDAI

The ESSDAI is a systemic disease activity index that was generated in 2009 in a cohort of 702 paper patients derived from 96 real patients. The ESSDAI includes 12 domains (constitutional, glandular, cutaneous, respiratory, renal, articular, muscular, peripheral and central nervous system, hematological, lymphoid, biological). Each domain is stratified into three or four levels according to the severity of manifestations [20]. The weights of each domain were obtained using the corresponding coefficient in a multiple regression model where PhGA on disease activity represented the dependent variable. Each physician is asked to rate only manifestations related to the disease activity and to avoid rating long-lasting stable clinical features in order to exclude rating of damage-related changes. The final score falls between 0 and, theoretically, 123, with 0 indicating no disease activity.

The ESSDAI has been validated in a prospective international cohort of 395 patients in 15 countries [22]. The aim of this study was to evaluate the psychometric properties (construct validity, responsiveness, and reliability) and compare them with those of previous measures. In the validation cohort, the ESSDAI score had higher correlation with PhGA than other previously proposed activity scores (Table 1), suggesting that the ESSDAI had better content and construct validity.

The reliability of the ESSDAI was assessed in a subgroup of 47 patients in the validation cohort. The intraclass correlation coefficient (ICC) was 0.96, 0.83, and 0.95 for ESSDAI, SSDAI, and SCAI, respectively, and thus reliability should be considered very good for all three systemic indices [22].

Preliminary data from the development study already showed that all the disease activity scores had a good sensitivity to change in patients whose disease activity had improved [23]. These data were confirmed in the validation study, showing that responsiveness of the three systemic indices, calculated as standardized response mean (SRM), ranged from moderate to large (Table 1) [22].

The MCII (Minimal Clinical Important Improvement) of the ESSDAI was estimated using a method based on the physician’s evaluation of change in disease activity. This parameter was defined as an improvement of the ESSDAI score by at least 3 points [24]. However, the ESSDAI detects changes more accurately than other indices (Table 1) [22].

After its release, the ESSDAI became a widely used instrument to assess systemic activity of pSS in clinical practice and in therapeutic trials. In a series of open label studies, the ESSDAI scores showed a certain degree of improvement after treatment with respect to the values at the patients’ enrollment [25,26,27,28]. Conversely, in randomized placebo-controlled studies where the ESSDAI was used as primary or secondary endpoint, this tool failed to show a significant difference between actively treated patients and the placebo group [29,30,31,32].

Different hypotheses have been proposed to explain these results. First of all, as in other autoimmune systemic disorders, activity flares may spontaneously recede, and this can also happen in the placebo-treated group. Second, the stratification of different domains in the ESSDAI was built using categorical items. Thus, some slight improvement in absolute values cannot be sufficient to induce a change in the ESSDAI domain category and therefore cannot be captured by this instrument. Finally, most of the enrolled patients in the RCTs had a low level of systemic activity and a too long disease duration to obtain a three-point reduction of the score, particularly in the presence of stable long-lasting clinical features [33].

Another point to be highlighted is that many ESSDAI domains are scored on the basis of the physician’s examination, which in multicenter trials can be influenced by the different cultural background and experience of the operators involved. A user guide of the ESSDAI, with a glossary precisely defining any included item, was also prepared to make it easier for less expert physicians to use this scoring system [34] and improve the interrater reliability in the data collection.

Moreover, a modified version of the ESSDAI, the so-called ClinESSDAI, where the biological domain has been excluded, was also released [35]. This modified version was proposed to allow a separate analysis of the potential correlations between variation of biological parameters and changes of specific clinical features in ESSDAI domains.

### 3.2. ESSPRI

The ESSPRI was developed in 2011 in a multicenter international cohort of 230 patients [21], with the purpose of assessing the main pSS-related symptoms, such as dryness, fatigue, and pain. Determination of the weight of symptoms was derived from the patients’ perspective by using a multiple linear regression model in which patient global assessment (PtGA) was the gold standard. The three ESSPRI domains were assessed by the patients by scoring 0 to 10 Likert scales for each of the domains. The patient is asked to rate how severe the symptom was during the past 2 weeks. The total score is the mean of the three domain scores and ranges from 0 to 10. The patient acceptable symptom state was defined as an ESSPRI score less than 5. The ESSPRI has been translated and validated in several languages: English, French, Danish, German, Greek, Dutch, Spanish, Italian, Japanese, Norwegian, Portuguese, Slovene, and Swedish.

Furthermore, the ESSPRI has been validated in the prospective international cohort of 395 patients from 15 countries [22]. The psychometric properties (construct validity, reliability, and responsiveness) were evaluated in this validation cohort and compared with those of previously described measures (SSI, Sicca Symptoms Inventory, and PROFAD, Profile Of Fatigue and Discomfort). The ESSPRI had a higher correlation with PtGA than that of other measures (Table 1). Moreover, the ESSPRI score showed a good correlation with both SSI (*r* = 0.59) and PROFAD (*r* = 0.68).

Reliability, assessed in a subgroup of 62 patients from a validation cohort, was considered very good for all the patient related outcomes (PROs). In this validation cohort, responsiveness of patient scores was low in patients experiencing improvement of their symptoms but was significantly higher for the ESSPRI compared with the SSI and PROFAD (Table 1) [22].

The MCII of the ESSPRI score is defined as an improvement of the score greater than or equal to 1 point or greater than or equal to 15% of the baseline value [24].

## 4. Systemic Disease Damage Measurements

Two scoring systems have been developed to assess general damage in pSS. The first one, the SS Disease Damage Index (SSDDI), was developed together with the SSDAI in the same Italian study. In its final version, the SSDDI scale included six domains and 15 items [16].

A modified version of SLICC/ACR-Dim, a damage index adopted in systemic lupus, was developed for specific use in pSS. Ocular and oral domains and eight systemic domains were included in a new damage index named SSDI (SS Damage Index). The results of a longitudinal study, aimed at verifying the validity of this index in assessing cumulated damage of patients with pSS, have been published [36].

## 5. Single Domain Assessment

### 5.1. Sicca Symptoms

It is well known that PROs for ocular and oral dryness in pSS poorly correlate with the real amount of glandular secretion [37]. In addition, subjective dryness is not correlated with the presence and severity of systemic features. However, subjective sicca symptoms greatly compromise the patient’s quality of life independent of the presence of active extra-glandular manifestations.

Ocular dryness can be assessed by the OSDI (Ocular Surface Disease Index) questionnaire [38]. In its validated version this instrument includes 12 items and three subscales, and test-retest reliability was good to excellent [39]. The OSDI proved to be valid since it was able to discriminate between normal, mild-to-moderate, and severe dry eye disease as defined by the physician’s assessment. Interrater and intrarater reliability ranged from good to excellent for the overall instrument and for each subscale [39].

Dry mouth symptomatology can be assessed by the Xerostomia Inventory (XI), an instrument composed of 11 items whose responses are summated to give a single score. The XI shows adequate content and construct validity but low correlation with unstimulated saliva flow [40].

Finally, responsiveness of the XI appears to be quite good, and a change of 6 or more scale points is considered clinically meaningful [40].

Composite instruments that are able to explore the cumulative impact of sicca symptoms in pSS have also been proposed. The Liverpool sicca index [41] and the subsequent SSI [42] explore in detail many facets of dryness, offering a complete view of sicca complaints from the patient perspective. SSI, also in its short form, contains items designed to assess oral, ocular, vaginal, and cutaneous dryness with a good discrimination power between patients with pSS and non-SS sicca controls.

### 5.2. Fatigue

Fatigue is one of the more invalidating symptoms in patients with pSS. Disease-specific PRO measures have been proposed to assess this complaint. The most commonly used questionnaires to measure fatigue are the Functional Assessment of Chronic Illness Therapy—Fatigue scale (FACIT) [43], the Fatigue Severity Scale (FSS) [44], and the Multidimensional Fatigue Inventory (MFI) [45]. These instruments include specific questions that investigate the different components of fatigue, such as somatic and mental aspects. Thus, all these questionnaires have demonstrated construct and content validity, but none have clearly shown superiority to a Visual Analogue Scale (VAS) in assessing change of fatigue after treatment. The simplest way of measuring fatigue is still the 10 cm VAS or a 0 to 10 Likert rating scale from “no fatigue” to “worst fatigue imaginable” [46].

### 5.3. Composite Instruments to Measure Subjective Symptoms in pSS

The PROFAD questionnaire [47] was derived from an initially broad questionnaire that captured key symptoms in patients with pSS, such as fatigue and pain. This Instrument was then combined with the SSI, and they are used together in a short form (SF) named the PROFAD-SSI-SF, developed in 2008 [48] with the purpose of assessing the main symptoms of patients with pSS. The key domains identified were dryness, physical and mental fatigue, limb pain, and vascular features attributed to Raynaud’s syndrome. A strong correlation between the SF and the previously proposed extended form was clearly demonstrated [47]. In its final form the PROFAD-SSI contains 19 items in eight domains: somatic fatigue (4 items), mental fatigue (2 items), arthralgia/limb pain (2 items), vascular dysfunction (Raynaud’s syndrome) (1 item), skin dryness (1 item), vaginal dryness (for women only) (1 item), ocular dryness (3 items), and oral dryness (5 items) [48].

The PROFAD-SSI was validated in a multicenter cohort in the United Kingdom in which psychometric properties were also evaluated. The reliability of PROFAD and SSI were separately investigated when they were first proposed. Construct validity of PROFAD was demonstrated by the fact that its separate domains strongly correlated with the corresponding domains of other similar instruments [47]. For instance, the PROF closely correlated with the MFI [47]. As to responsiveness, PROFAD has not so far demonstrated significant improvement after any therapeutic intervention. However, it has been proposed that the MCII of the PROFAD-SSI-SF should be defined as an improvement of greater than or equal to 1 point or greater than or equal to 15% of the baseline value.

### 5.4. Quality of Life

Disease-specific instruments applied to assess the specific complaints of pSS (e.g., dryness, chronic pain, and physical and mental fatigue) usually work better than a generic questionnaires for this purpose. A specific instrument has also been created to assess health-related quality of life (HRQoL) in pSS (pSS-HRQoL) [49]. Construct validity of this questionnaire was demonstrated by the strong correlation found with ESSPRI. Intrarater reliability of the pSS-QoL was also very good [49]. However, the Short-Form 36 (SF36) has been the most widely used tool to investigate this domain in pSS.

A recent meta-analysis of the studies in which the SF36 was used to assess HRQoL in pSS demonstrated that the disease has a significant negative impact on HRQoL, especially on the physical function domains, although pSS also affects patients from the psychological points of view [50].

### 5.5. Objective Measurements of Salivary Gland Function

Measurement of salivary flow is the most common way to assess salivary gland function. The collection of saliva in a graduated tube is usually performed without stimulation in a given time. The physiological unstimulated whole salivary flow (UWSF) is around 0.3–0.4 mL/min. It is considered abnormal when it is equal or below 0.1 mL/min [51]. This threshold is recognized as having a diagnostic value in distinguishing patients with pSS, and it has been included as a separate item in the classification criteria for this disorder [52,53]. UWSF is considered more sensitive than stimulated salivary flow for diagnostic purposes [54]. In some therapeutic trials with rituximab, it has been shown that salivary flow may improve after active treatment, particularly in those patients with residual pre-treatment capability of producing saliva after stimulation [55,56].

### 5.6. Objective Assessment of Salivary Gland Structure

Major salivary gland ultrasound (SGUS) examination is currently the most popular method used to evaluate pSS-related anatomical changes. Although this methodology is largely used in clinical practice, an agreement on how to perform it and evaluate the observed abnormalities has not been reached so far, and therefore, reliability of this imaging assessment has not been precisely defined [57]. The presence of hypoecogenic areas in the glands is considered the most specific finding observed in patients with pSS [58].

Despite the potential usefulness of SGUS in the diagnosis of pSS, its value in assessing disease activity and disease progression needs to be established. Different SGUS scores have been proposed, and some of them seem to correlate with objective salivary gland function, such as UWSF rates. In addition, some studies have demonstrated some degree of correlation between SGUS scores and parameters indicative of disease activity, such as ESSDAI scores, IgG levels, and rheumatoid factor levels. Conversely, it has been suggested that hypoechogenic areas reflect the level of damage of the glands [59].

### 5.7. Histopathologic Assessment of Salivary Gland Features

Minor salivary gland biopsy (MSGB), performed in the middle of the lateral part of the inferior lip, is the most commonly used and an almost completely safe procedure to obtain salivary tissue to be analyzed for diagnostic and investigative purposes [60]. In order to improve the reliability of MSGB, an agreement on the precise methodology to perform it and analyze the obtained tissue was reached by a board of experts [61]. Few data exist on the progression and evolution of the MSGB changes in pSS. Thus, the advantages of repeating biopsies in longitudinal studies and therapeutic trials are still debated. Ethical concerns have been raised about performing serial biopsies, particularly on patients treated with placebo. The demonstration of an improvement in biopsy scores in pilot studies, where a placebo group is excluded, could support the idea of introducing MSGB as an additional end point in future studies [61].

From this point of view, a prospective follow up study conducted in Italy [62] is of particular interest. In this study, tissue from two MSGBs was obtained at the time of enrollment and after 120 weeks from patients treated with conventional disease modifying anti-rheumatic drugs (DMARDs) and patients treated with rituximab. A strong reduction in the focus score and number of germinal-like centers was found in the second biopsies only in rituximab-treated patients.

### 5.8. Objective Assessment of Lachrymal Function

Two types of measures are commonly used for the objective evaluation of ocular dryness: those that quantify the tear production, such as Schirmer’s test, and those that assess the consequence of tear film abnormalities, such as ocular surface dye scores.

Schirmer’s test is performed by the insertion of paper strips in the inferior eyelid for 5 min. The paper is then removed, and the length of paper strip wetted by the tears produced is measured. The test is considered indicative for a diagnosis of pSS when the moisture is less than or equal to 5 mm in 5 min [63]. Despite its poor sensitivity, Schirmer’s test is widely used in clinical practice since it is feasible and easily done by any physician, without requiring the intervention of an ophthalmologist. It has also been included among the items composing the most recent American College of Rheumatology/European League against Rheumatism classification criteria [53].

The break-up time (BUT) test is another easy and fast method used to assess the stability of tear film. The test consists of the measurement of the time prior to the appearance of the first dry spot in the fluorescein-stained tear film on the cornea surface, observed by using a slit lamp apparatus [64]. BUT is rarely used in pSS because of its poor reliability and specificity. Apart from tear production, the stability of tear film depends on multiple factors, such as increased evaporation rate, tear hyperosmolarity, and the presence of any type of inflammatory process [64].

By contrast, quantification of ocular surface staining remains the mainstay for objective dry eye assessment in pSS. The two most commonly used staining evaluation methods for pSS are van Bijsterveld’s scoring method [65] and the Ocular Staining Score (OSS) [66]. These two tools are very similar and strongly correlated to each other.

In van Bijsterveld’s method, the intensity of lissamine green staining is qualitatively scored in the temporal and nasal conjunctival zones and in the cornea. In each of the three zones, the score ranges from 0 to 3, giving a maximum score of 9. A score equal or greater that 4 is considered diagnostic for pSS [65].

**Figure 1:**
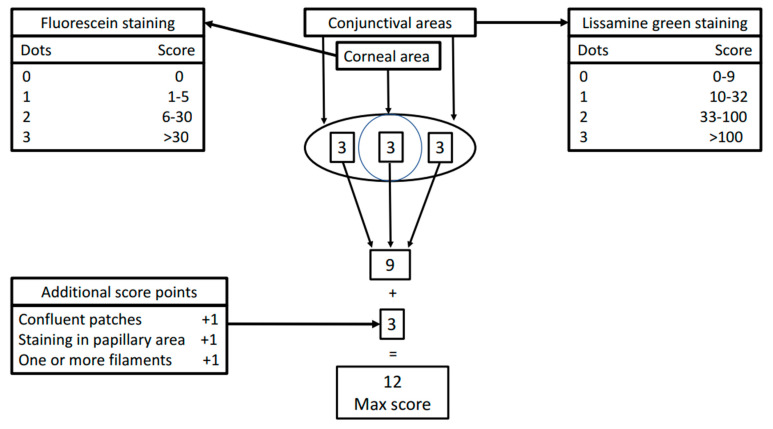
Ocular Staining Score (OSS): a graphical representation of the scoring method of this ophthalmologic test for the assessment of epithelial lesions on ocular surface in pSS.

The OSS was developed in 2010 by the Sjögren’s International Collaborative Clinical Alliance research group [66]. The OSS includes two domains: the corneal fluorescein staining pattern and the conjunctival lissamine green staining pattern. The cornea is examined and scored from 0 to 3 according to the number of the counted punctate epithelial erosions. A score 0 is given in the absence of any erosion. Scores 1, 2, and 3 are given in the presence of 1 to 5, 6 to 30, and more than 30 dots, respectively. Nasal and temporal conjunctivae are graded separately, with a maximum score of 3 for each area or a total maximum score of 6 for each eye. Grade 0 in each conjunctival area is defined as 0 to 9 dots, grade 1, 10 to 32 dots, grade 2, 33 to 100 dots, and grade 3, more than 100 dots (Figure 1).

Additional points are added if the punctate erosions occurred in the central portion of the cornea, in the presence of one or more filaments, and one or more area of confluent staining on the cornea (Figure 1).

The total OSS for each eye is the summation of the fluorescein score for the cornea and the lissamine green scores for the nasal and temporal bulbar conjunctivae. Thus, the maximum possible score for each eye is 12. An OSS higher than 5 in at least one eye is considered indicative of a diagnosis of pSS [53].

The reliability of the OSS has been assessed, but interrater numerical agreement was very poor, since the score can rapidly change over time. This fact may suggest that the OSS could be used as a very sensitive end point in therapeutic trials. However, there are very few data on the ability of the OSS to detect significant change over time and, until now, the MCII has still not been defined.

## 6. General Considerations

All the instruments analyzed in previous paragraphs are currently in use in the management of patients with pSS. Some of them are also included among the items selected for the definition of the ACR-EULAR classification criteria for this disease and then commonly performed in any patients at least at the moment of diagnostic definition. Instruments devoted to the precise assessment of some other domains (activity, fatigue, sicca symptoms, pain, quality of life) are not in common use in clinical practice but are considered as mandatory when a single domain has to be precisely assessed in the course of randomized clinical trials. Some limitations have emerged regarding the performance of some of them in terms of reliability and sensitivity to change when used in this context (see previous comments on ESSDAI).

## 7. Future Direction: Use of Composite Indices for More Complete Assessment of pSS

pSS is a multi-faceted disorder with a heterogeneous mode of presentation in different groups of patients. Systemic involvement, which is present only in some patients, can be due to inflammatory infiltration of different non-glandular epithelia or microvascular changes due to local pathological deposition of autoantibodies or immune complexes [67]. Thus, the underlying pathological mechanisms may substantially differ in these subsets of patients. Furthermore, almost all the patients complain of sicca symptoms that are related to long lasting and progressive glandular involvement, often leading to irreversible glandular damage. Consequently, early assessment and treatment of glandular involvement may prevent late damage and severe sicca complaints which, together with fatigue, mostly influence the patients’ quality of life [67,68].

The extreme heterogeneity of clinical features in pSS implies the need for different treatment targets. Thus, composite end points capable of combining the assessment of systemic disease activity, subjective symptoms, glandular function, and serological parameters could be more suitable for appreciating the efficacy of any new treatment on the different aspects of the disease.

An interesting multinational project, whose acronym is “NECESSITY”, is ongoing. Its aim is to develop and validate a new composite end point, the SS Tool for Assessing Response (STAR) [69].

Another composite end point has been developed by the analysis of data from rituximab and abatacept trials. Its acronym is CRESS (Composite and Relevant Endpoints in SS). This includes five instruments that are able to assess different disease domains, i.e., systemic disease activity (ClinESSDAI), patient-reported symptoms (ESSPRI), tear gland function (Schirmer’s test and OSS), salivary function (UWSF and salivary US examination), and serological items (rheumatoid factor and immunoglobulin G) [70].

The use of these proposed composite instruments may open a new era in the outcome evaluation of patients with pSS, giving the possibility of a contemporary but differentiated assessment of different domains on the wide spectrum of the disease. Of course, it will be necessary to validate the proposed composite indices in future studies.

## Figures and Tables

**Table 1:** Construct validity, reliability, and sensitivity to change (responsiveness) of the main outcome measures used for the assessment of patients with pSS [22].

	Construct Validity, Correlation Coefficient *	Reliability, ICC ° (CI 95%)	Responsiveness, SRM ^ (CI 95%) in Improved Patients
*Physician-oriented outcome measures*	versus PhGA	*n* = 47	*n* = 62
ESSDAI	0.59	0.96 (0.89 to 0.98)	−0.72 (−0.97 to −0.57)
SSDAI	0.34	0.83 (0.56 to 0.94)	−0.82 (−1.06 to −0.62)
SCAI	0.32	0.95 (0.85 to 0.98)	−0.69 (−0.95 to −0.47)
*Patient-oriented outcome measures*	versus PtGA	*n* = 62	*n* = 95
ESSPRI	0.70	0.94 (0.89 to 0.97)	−0.37 (−0.60 to −0.17)§
SSI	0.65	0.86 (0.77 to 0.91)	−0.04 (−0.26 to +0.23)
PROFAD	0.58	0.92 (0.87 to 0.96)	−0.16 (−0.35 to +0.06)

* by Spearman’s correlation. ° ICC = interclass correlation coefficient that was used to assess interrater reliability. ^ SRM = Standardized Response Mean. This is calculated by dividing the *mean* change by the standard deviation of the change. The result is considered moderate when ≥0.50 and <0.80, and large when ≥0.80. SRM of physician-oriented outcome measures was lower in stable and worsened patients (not reported here). § SRM values significantly higher than those observed in SSI (*p* = 0.006) and PROFAD (*p* = 0.049). CI, confidence intervals. For other abbreviations, see text.
